# Entropy-Based Algorithms for Signal Processing

**DOI:** 10.3390/e22060621

**Published:** 2020-06-04

**Authors:** Gwanggil Jeon, Abdellah Chehri

**Affiliations:** 1 Department of Embedded Systems Engineering, College of Information Technology, Incheon National University, 119 Academy-ro, Yeonsu-gu, Incheon 22012, Korea; 2 Department of Applied Sciences, University of Quebec in Chicoutimi (UQAC), Chicoutimi, QC G7H 2B1, Canada; achehri@uqac.ca

**Keywords:** multichannel imaging, sensor, dynamic range, modeling of signal processing, compression approach, entropy-based video coding, prediction and redundancy, noise removal

## 1. Introduction

Entropy, the key factor of information theory, is one of the most important research areas in computer science. Entropy coding informs us of the formal limits of today’s storage and communication infrastructure. Over the last few years, entropy has become an important trade-off measure in signal processing. Entropy measures especially have been used in image and video processing by applying sparsity and are able to help us to solve several of the issues that we are currently facing. As the daily produced data are increasing rapidly, a more effective approach to encode or compress the big data is required. In this sense, applications of entropy coding can improve image and video coding, imaging, quality assessment in agricultural products, and product inspection, by applying more effective coding approaches.

In light of these and many other challenges, a Special Issue of Entropy-Based Algorithms for Signal Processing has been dedicated to address the current status, challenges, and future research priorities for the entropy of signal processing.

## 2. Themes of This Special Issue

Starting from the above considerations, this Special Issue aims to investigate the impact of the adoption of advanced and innovative entropy-based algorithms in signal processing applications, including the ones that take advantage of recent big data, compression, multichannel, sensor and prediction techniques. This edition of the Special Issue is focused primarily on signal processing for image and video applications with particular emphasis to stream processing and imaging platforms. This issue is intended to provide a highly recognized international forum to present recent advances in entropy. We welcomed both theoretical contributions as well as papers describing interesting applications. Papers were invited for this Special Issue considering aspects of this problem, including:

- Multichannel imaging;
- Sensor size, channel number, dynamic range;
- Modeling of signal processing;
- Compression approach;
- Entropy-based video coding;
- Prediction and redundancy for video coding;
- Noise removal approach;
- GPU-based methods for signal processing;
- Signal quality assessment.

After review, a total of 10 papers have been accepted for publication in this issue.

### 2.1. Models

In the contribution by Ahmed et al. [1], “Two-Dimensional Permutation Vectors’ Code for Optical Code Division Multiple Access Systems,” authors presented a new algorithm to generate two-dimensional permutation vectors’ code for an incoherent optical code division multiple access (OCDMA) system to suppress multiple access interference and system complexity. The proposed code design approach is based on a wavelength-hopping time-spreading technique for code generation. All possible combinations of PV code sets were attained by employing all permutations of the vectors with the repetition of each vector weight times. Furthermore, a 2D-PV code set was constructed by combining two code sequences of the 1D-PV code. The transmitter-receiver architecture of the 2D-PV code-based WHTS OCDMA system is presented. Their results indicate that the 2D-PV code provides increased cardinality by eliminating phase-induced intensity noise effects, and multiple user data can be transmitted with a minimum likelihood of interference. Simulation results validated the proposed system for an agreeable bit error rate of $10^{-9}$.

The contribution by Shan and Fang [2], “A Cross Entropy Based Deep Neural Network Model for Road Extraction from Satellite Images,” proposes a deep convolutional neural network model with an encoder-decoder architecture to extract a road network from satellite images. The authors employ ResNet-18 and atrous spatial pyramid pooling technique to tradeoff between the extraction precision and running time. A modified cross-entropy loss function is proposed to train their deep model. A PointRend algorithm is used to recover a smooth, clear, and sharp road boundary [3]. The augmented DeepGlobe dataset is used to train their deep model, and the asynchronous training method is applied to accelerate the training process. Five satellite images covering Xiaomu village are taken as input to evaluate their model. The proposed E-Road model has fewer number of parameters and shorter training time. Their experimental results show that the E-Road outperforms other state-of-the-art deep models with 5.84% to 59.09% improvement, and can give accurate predictions for the images with a complex environment.

In the contribution by Bao et al. [3], “A Multiple-Input Multiple-Output Reservoir Computing System Subject to Optoelectronic Feedbacks and Mutual Coupling,” a multiple-input multiple-output reservoir computing (RC) system is proposed. The system is composed of multiple nonlinear nodes (Mach–Zehnder modulators) and multiple mutual-coupling loops of optoelectronic delay lines. Each input signal is added into every mutual-coupling loop to implement the simultaneous recognition of multiple route signals, which results in the signal processing speed improving improvements and the number of routes increasing. As an example, the four-route input and four-route output RC is simultaneously realized by numerical simulations. Their results show that this type of RC system can successfully recognize the four-route optical packet headers with 3-bit, 8-bit, 16-bit, and 32-bit, and four-route independent digital speeches. When the white noise is added to the signals such that the signal-to-noise ratio (SNR) of the optical packet headers and the digital speeches are 35 and 20 dB respectively, the normalized root mean square errors (NRMSEs) of the signal recognition are all close to 0.1. The word error rates (WERs) of the optical packet header recognition are 0%. The WER of digital speech recognition is 1.6%. The eight-route input and eight-route output RC is also numerically simulated. The identification of the eight-route 3-bit optical packet headers is implemented. The parallel processing of multiple-route signals and the high recognition accuracy are implemented by this proposed system.

The purpose of the contribution by Urniezius and Survyla [4], “Identification of Functional Bioprocess Model for Recombinant E. Coli Cultivation Process,” is to introduce an improved Luedeking–Piret model that represents a structurally simple biomass concentration approach. The developed routine provides acceptable accuracy when fitting experimental data that incorporates the target protein concentration of Escherichia coli culture BL21 (DE3) pET28a in fed-batch processes. Their work presents system identification, biomass, and product parameter fitting routines, starting from their origin to the entropy-related development, characterized by robustness and simplicity. A single tuning coefficient allows for the selection of an optimization criterion that serves equally well for higher and lower biomass concentrations. The paper’s idea is to demonstrate that the use of fundamental knowledge can make the general model more common for technological use than to a sophisticated artificial neural network. Experimental validation of their proposed model involved data analysis of six cultivation experiments compared to 19 tests used for model fitting and parameter estimation. 

### 2.2. Performance Improvement

Compound fault diagnosis is challenging due to the complexity, diversity, and non-stationary characteristics of complex mechanical faults. In the contribution by Tang and Tian [5], “Compound Fault Diagnosis of Rolling Bearing Based on Singular Negentropy Difference Spectrum and Integrated Fast Spectral Correlation,” a novel compound fault separation method based on singular negentropy difference spectrum (SNDS) and integrated fast spectral correlation (IFSC) is proposed. Firstly, the original signal was de-noised by SNDS, which improved the noise reduction effect of the singular difference spectrum by introducing negative entropy. Secondly, the de-noised signal was analyzed by a fast spectral correlation. Finally, IFSC took the fourth-order energy as the index to determine the resonance band and separate the fault features of different single faults. Their proposed method was applied to analyze the simulated compound signals and the experimental vibration signals. Their results show that the proposed method has an excellent performance in the separation of rolling bearing composite faults. 

With the wide applications of three-dimensional (3D) meshes in intelligent manufacturing, digital animation, virtual reality, digital cities, and other fields, more processing techniques are being developed for 3D meshes. These techniques, including watermarking, compression, and simplification, which will inevitably lead to various distortions. Therefore, how to evaluate the visual quality of the 3D mesh is becoming a crucial problem, and it is necessary to design practical tools for blind 3D mesh quality assessment. In the contribution by Lin et al. [6], “Blind Mesh Assessment Based on Graph Spectral Entropy and Spatial Features,” authors propose a new blind mesh quality assessment method based on graph spectral entropy and spatial features, called as BMQA-GSES. The 3D mesh can be represented as graph signal. In the graph spectral domain, the Gaussian curvature signal of the 3D mesh is firstly converted with graph Fourier transform (GFT). Then the smoothness and information entropy of amplitude features are extracted to evaluate the distortion. In the spatial domain, four well-performing spatial features are combined to describe the concave and convex information and structural information of 3D meshes. All the extracted features are fused by the random forest regression to predict the objective quality score of the 3D mesh. Based on their assumption, experiments were performed successfully on public databases. The obtained results show that the proposed BMQA-GSES method provides a good correlation with human visual perception and competitive scores compared to state-of-art quality assessment methods. 

A continuous path performed by the hand in a period of time is considered for gesture recognition. Dynamic gesture recognition is a complex topic since it spans from the conventional method of separating the hand from the surrounding environment to searching for the fingers and palm. The contribution by Alejo and Funes [7], “Recognition of a Single Dynamic Gesture with the Segmentation Technique HS-ab and Principle Components Analysis (PCA),” proposes a strategy of hand recognition using a PC webcam, a segmentation technique, and pre-processing of images to reduce noise and a classifier such as principal components analysis for the detection and tracking of the hand of the user. Their results show that the segmentation technique HS-ab and the method PCA are robust in the execution of the system. However, there are various conditions such as illumination, speed, and precision of the movements. For this reason, suitable extraction and classification of features allow the gesture’s location. The system was tested with a database of training images and had a 94.74% accuracy.

### 2.3. Applications

Image fusion is an efficient technique that can be applied in many fields, such as medicine, remote sensing, and surveillance.

In the contribution by Liu et al. [8], “Entropy-Based Image Fusion with Joint Sparse Representation and Rolling Guidance Filter,” an image fusion method using multi-scale decomposition and joint sparse representation is introduced. There are five steps in this work. First, joint sparse representation is applied to decompose two source images into a common image and two innovation images. Second, two initial weight maps are generated by filtering the two source images separately. Third, weight maps are obtained by joint bilateral filtering according to the initial weight maps. Fourth, the multi-scale decomposition of the innovation images is performed through the rolling guide filter. Finally, Lastly, the final weight maps are used to generate the fused innovation image. The fused innovation image and the common image are combined to generate the ultimate fused image. The experimental results show that their method’s average metrics are: mutual information—5.3377, feature mutual information—0.5600, normalized weighted edge preservation value—0.6978, and nonlinear correlation information entropy—0.8226. Their approach can achieve better performance compared to the state-of-the-art methods in visual perception and objective quantification. 

In multi-modality image fusion, source image decomposition, such as multi-scale transform, is a necessary step and also widely used. However, when MST is directly applied to decompose source images into high- and low-frequency components, the corresponding decomposed components are not precise enough for the following infrared-visible fusion operations. The contribution by Huang et al. [9], “A Novel Infrared and Visible Image Information Fusion Method Based on Phase Congruency and Image Entropy,” proposes a non-subsampled contourlet transform based decomposition method for image fusion, by which source images are decomposed to obtain corresponding high- and low-frequency sub-bands. Unlike MST, the obtained high-frequency sub-bands have different decomposition layers, and each layer contains different information. To get a more informative, a fused high-frequency component, maximum absolute value, and pulse coupled neural network fusion rules are applied to different sub-bands of high-frequency components. Activity measures, such as phase congruency, local measure of sharpness change, and local signal strength, are designed to enhance the detailed features of fused low-frequency components. The fused high- and low-frequency components are integrated to form a fused image. Their experiment results show that the fused images obtained by the proposed method achieve good performance in clarity, contrast, and image information entropy. 

Image recovery from compressive sensing measurement data, especially noisy data, has always been challenging due to its inherent ill-posed nature. Thus, to seek a domain where a signal can exhibit a high degree of sparsity and to design an effective algorithm has drawn more attention. 

Among various sparsity-based models, structured or group sparsity often leads to more powerful signal reconstruction techniques. In the contribution by Xie et al. [10], “An Entropy-Based Algorithm with Nonlocal Residual Learning for Image Compressive Sensing Recovery,” authors propose a novel entropy-based algorithm for CS recovery to enhance image sparsity through learning the group sparsity of residual. 

To reduce the residual of similar packed patches, the group sparsity of residual is described by a Laplacian scale mixture model. Therefore, each singular value of the residual of similar packed patches is modeled as a Laplacian distribution with a variable scale parameter, to exploit the benefits of high-order dependency among sparse coefficients. Due to the latent variables, the maximum a posteriori estimation of the sparse coefficients cannot be obtained. Thus, the authors design a loss function for the expectation-maximization method based on relative entropy. In the EM iteration frame, the sparse coefficients can be estimated with the denoising-based approximate message passing algorithm. Their experimental results have shown that the proposed algorithm can significantly outperform existing CS techniques for image recovery.

## 3. Conclusions

The articles presented in this Special Issue provide insights in fields related to signal processing using entropy-based algorithms, including models, performance evaluation and improvements, and application developments. We wish the readers can benefit from insights of these papers, and contribute to these rapidly growing areas. We also hope that this Special Issue would shed light on major developments in the area of entropy and attract attention by the scientific community to pursue further investigations leading to the rapid implementation of these technologies.

## References

[1] Ahmed H.Y., Zeghid M., A.Imtiaz W., Sharma T., Chehri A., Fortier P. (2020). Two-Dimensional Permutation Vectors’ (PV) Code for Optical Code Division Multiple Access Systems. Entropy.

[2] Shan B., Fang Y. (2020). A Cross Entropy Based Deep Neural Network Model for Road Extraction from Satellite Images. Entropy.

[3] Bao X., Zhao Q., Yin H. (2020). A Multiple-Input Multiple-Output Reservoir Computing System Subject to Optoelectronic Feedbacks and Mutual Coupling. Entropy.

[4] Urniezius R., Survyla A. (2019). Identification of Functional Bioprocess Model for Recombinant *E. Coli* Cultivation Process. Entropy.

[5] Tang G., Tian T. (2020). Compound Fault Diagnosis of Rolling Bearing Based on Singular Negentropy Difference Spectrum and Integrated Fast Spectral Correlation. Entropy.

[6] Lin Y., Yu M., Chen K., Jiang G., Chen F., Peng Z. (2020). Blind Mesh Assessment Based on Graph Spectral Entropy and Spatial Features. Entropy.

[7] Contreras Alejo D.A., Gallegos Funes F.J. (2019). Recognition of a Single Dynamic Gesture with the Segmentation Technique HS-ab and Principle Components Analysis (PCA). Entropy.

[8] Liu Y., Yang X., Zhang R., Albertini M.K., Celik T., Jeon G. (2020). Entropy-Based Image Fusion with Joint Sparse Representation and Rolling Guidance Filter. Entropy.

[9] Huang X., Qi G., Wei H., Chai Y., Sim J. (2019). A Novel Infrared and Visible Image Information Fusion Method Based on Phase Congruency and Image Entropy. Entropy.

[10] Xie Z., Liu L., Yang C. (2019). An Entropy-Based Algorithm with Nonlocal Residual Learning for Image Compressive Sensing Recovery. Entropy.

